# Byte into sustainability: a scoping review of digital food environment attributes that shape consumers’ sustainability perceptions, attitudes, intentions, and behaviours

**DOI:** 10.1186/s12966-025-01832-6

**Published:** 2025-10-27

**Authors:** Hannah Boen, Louise Glenisson, Lotte Hallez, Tim Smits

**Affiliations:** Media, Information & Persuasion Lab, KU Leuven, Leuven, Belgium

**Keywords:** Digital food environment, Scoping review, Digital media, Sustainable food, Consumers, Intentions, Perceptions, Attitudes, Behaviours

## Abstract

**Background:**

Current food systems harm both planetary and human health. Meanwhile, our food choices are increasingly influenced by the digital food environment: spaces where digital actors (such as influencers) engage in food-related activities (like sharing content) across various platforms (for example, on social media). The digital food environment offers potential to promote more sustainable diets in several ways; for instance, influencers may encourage followers to reduce meat consumption, and supermarket apps can highlight the carbon footprint of different products. However, research on how specific features of the digital food environment, referred to here as digital attributes, affect sustainable food outcomes remains scattered. This scoping review aims to address this gap by: (1) mapping the scope and volume of current research on the relationship between the digital food environment and consumers’ sustainable food outcomes; and (2) identifying digital attributes that influence these outcomes. Consumers’ sustainable food outcomes are defined as consumers’ perceptions, attitudes, intentions, or behaviours related to social, economic, or environmental aspects of food sustainability.

**Methods:**

A systematic search was conducted in Web of Science, PubMed, and Scopus for peer-reviewed studies published between 2014 and 2024, following PRISMA-ScR guidelines. Studies were included if they examined the relationship between digital attributes and sustainable food outcomes. A total of 57 articles met the inclusion criteria.

**Results:**

Publications focusing on the relationship between the digital food environment and sustainable food outcomes have been on an upward trend since 2014. Most studies originate from high-income countries (46.9%) and are predominantly quantitative (91%), focusing on general adult populations (88.3%). Across five types of digital media, 86 unique digital attributes were identified. E-commerce emerged as the most frequently studied medium (57.9%), with attributes primarily related to informative messaging and platform design. Social media followed (28.1%). Social media attributes were mainly centred on social media marketing. Other digital media, such as documentaries (7%), were explored far less.

**Conclusion:**

This review highlights growing academic interest in the links between digital attributes and consumers’ sustainable food outcomes. Future research should broaden its scope to include other digital media, consumer experiences, and underrepresented populations.

**Supplementary Information:**

The online version contains supplementary material available at 10.1186/s12966-025-01832-6.

## Background

Current food systems negatively impact the health of the planet and its inhabitants by depleting natural resources, polluting the environment, contributing to undernourishment, exacerbating obesity, and marginalising small food actors [1–4]. In response to these challenges, policymakers are increasingly prioritising the transition to sustainable food systems that benefit people, the planet, and the economy [3], as evidenced by initiatives such as the European Green Deal [5] and the United Nations Sustainable Development Goals [6]. In food sustainability legislation, there is also a growing emphasis on the role of digital media (e.g., e-commerce websites, social media platforms, documentaries) in promoting sustainable diets [7].

Since the 2000s, digitisation has permeated all aspects of society, including the food industry [8, 9]. With the rise of the internet, food has gained a prominent online presence: consumers now order food online, encounter food content on their social media feeds, and browse the web for food-related information. As such, food choices are influenced not only by the physical food environment, but also by the digital food environment, which can be viewed as “an expansion and augmented experience of the physical food environment mediated by digital technologies” ([8] p. 12) where digital actors (such as influencers) engage in food-related activities (like sharing content) across various platforms (for example, on social media).

The term digital food environment was first introduced by Granheim [10] in 2019, sparking its uptake in both research and policy. Several review articles have since emerged (e.g., [9, 11, 12]) and the World Health Organisation [13] has dedicated a research stream to the concept. However, most existing research on the digital food environment focusses on its relationship with healthy eating, while sustainable eating has received far less attention.

The digital food environment holds considerable potential to support sustainable diets. For example, online communities can share advice on home food production, while e-commerce platforms may offer filters that sort products by sustainability certifications. However, existing research on elements of the digital food environment that could positively influence consumers’ sustainable food outcomes, referred to here as digital attributes, is fragmented and limited in scope. Most existing studies focus narrowly on one or two specific digital attributes, for example, the effect of displaying ecoscores in an online supermarket. Far less attention has been given to how such attributes sit within the broader digital food environment. Consequently, no comprehensive overview currently exists of the full spectrum of digital attributes examined in the literature. A structured, macro-level review is therefore required to map these attributes, situate them within the wider digital food environment, and summarise the current state of research.

To address this gap, we conducted a scoping review. To encompass both the ‘digital factors’ examined in quantitative research and the ‘digital drivers and inhibitors’ highlighted in qualitative studies, we adopted the inclusive term ‘digital attributes’ to refer to features of the digital food environment. The scoping review approach was chosen due to the scarcity of existing reviews on this topic, the multifaceted nature of the ‘digital food environment’, and the dual aim of this study. The first objective was to map the scope and volume of current evidence on the relationship between the digital food environment and consumers’ sustainable food outcomes. The second aim was to identify digital attributes that shape these outcomes. We define consumers’ sustainable food outcomes as sustainable food perceptions, attitudes, intentions or behaviours related to one of three dimensions of sustainability (social, economic, or environmental sustainability), outlined by Food and Agriculture Organization of the United Nations [14].

## Method

This scoping review was conducted as part of a series of systematic reviews aimed at uncovering social, individual, digital, and physical drivers and inhibitors of sustainable food outcomes. The protocol for these reviews was preregistered in the International Prospective Register for Systematic Reviews (PROSPERO) (case number: CRD42024538965). The protocol for this specific scoping review was preregistered in Open Science Framework [15]. We followed the PRISMA-ScR guidelines (Preferred Reporting Items for Systematic reviews and Meta-Analyses extension for Scoping Reviews) [16]. The PRISMA-ScR checklist is included in the supplementary materials. As the study relied exclusively on secondary literature sources, ethical approval was not required.

### Eligibility criteria

In order to sample relevant studies relating to the digital food environment and sustainable food outcomes, several inclusion and exclusion criteria were formulated.

Studies had to be English-language, peer-reviewed journal articles published between 2014 and 2024. This timespan was chosen due to the notable increase in publications on sustainable food since 2014 [17]. Eligible studies encompassed qualitative, quantitative, or mixed-method research, conducted either in laboratory, natural or online settings. Review articles, meta-analyses, conference abstracts, dissertations, books, and book chapters were excluded to maximize quality.

Eligible studies needed to investigate at least one attribute of the digital food environment as an independent variable, moderator or mediator. They also had to outline at least one sustainable food outcome (i.e., perceptions, attitudes, intentions or behaviours) related to one of three dimensions of sustainability (social, economic, or environmental sustainability), outlined by Food and Agriculture Organization of the United Nations [14]. Studies on genetically modified organisms (GMOs) were excluded due to differing perspectives on their sustainability [18].

Eligible studies could include participants of any gender, age, nationality, socio-economic status, or educational level. However, studies were excluded if more than half of the participants had a specific disease or disorder (e.g., diabetes, eating disorder) or if the majority of participants belonged to a clinical population, as these groups may have dietary restrictions or medical advice influencing their eating behaviours. Similarly, studies examining behaviours related to alcohol or drug use were also excluded.

### Search strategies and data charting

Because this paper is part of a larger group of reviews, a single comprehensive search string was created (see supplementary materials). The search string consisted of ten components for which the keywords were derived from relevant literature (see Table 1). Two of the components (psychological state factors and physical food environment) were added because they were relevant for related reviews in the project (see [19] and protocol with PROPSERO case number: CRD42024538965). Prior to the initial screening, the search string was tested and revised to yield an exhaustive yet relevant list of studies.

**Table 1:** Search string components

Component	Keyword theme	Examples of keywords
1	Year of publication	2014-now
2	Population	Human, consumer, adult, teen, women, young, customer, family, minority
3	Consumers’ psychological state factors	Character, emotional state, personal conviction
4	Physical food environment	Store, retail, supermarket, market, restaurant
5	Digital food environment	Television, movie, series, internet, site, social media, online, digital, movie, food delivery platform, apps, advergame, UGC, e-commerce, podcast, blog
6	Sustainability	Zero-waste, eco-friendly, green, plant-based, sustainable, organic
7	Food	Food, diet, snack, drink, meal, fruit
8	Outcomes	Behaviour, eat, intake, choice, perception, attitude
9	Exclusion criteria	Diabetes, cancer, pregnant, antiviral
10	Language	English

**Fig. 1:**
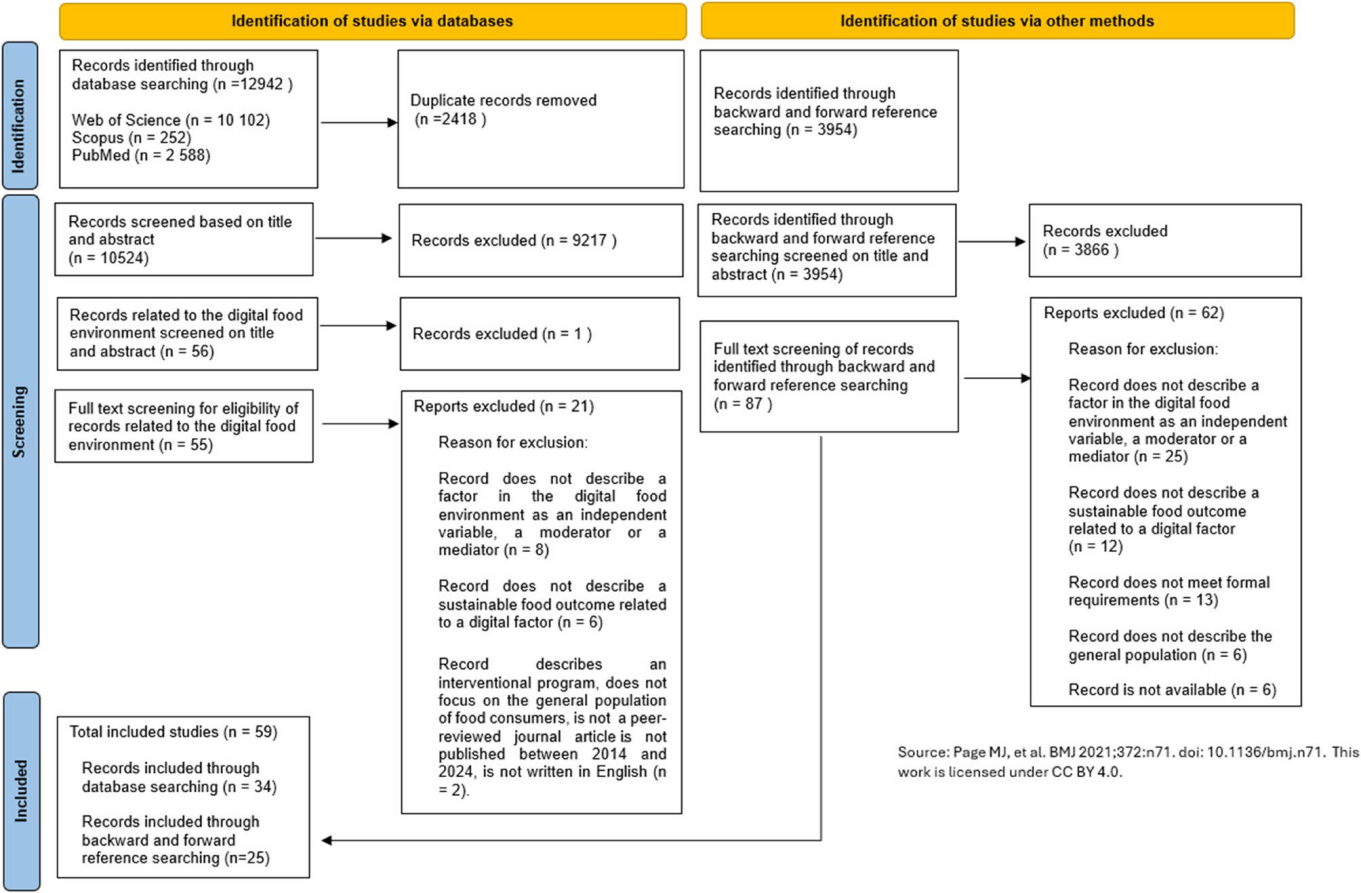
Flowchart of selection process

The initial search was conducted across three electronic databases: Web of Science, PubMed, and Scopus. Together these databases provide comprehensive, high-quality, and interdisciplinary coverage, capturing health, social, environmental, and technological research relevant to the digital food environment and sustainable food outcomes. This search identified 12,942 articles, from which 2,418 duplicates were removed using SR Accelerator software [20]. The remaining studies were imported into Zotero, an online reference management tool. One researcher (L.G.) screened all titles and abstracts against the predefined eligibility criteria for the study reported here, narrowing the sample to 56 articles. A second researcher (H.B.) cross-checked all titles and abstracts, reducing the sample to 55 papers. H.B. then conducted a full-text screening, selecting 33 studies. Subsequently, H.B. performed backward and forward reference searches on these studies, identifying 87 additional papers. After iterative full-text screening, 24 of these were retained, resulting in a final sample of 57 studies. Throughout the screening process, H.B. regularly consulted other colleagues (L.G., L.H., T.S.) when uncertainties arose. Figure 1 summarises the screening process.

H.B. conducted the data extraction for the final sample using a standardised form that had been tested across all reviews preregistered in PROSPERO (CRD42024538965). The form captured variables including title, author(s), year of publication, journal, theoretical frameworks, methodological characteristics, sample details, research questions, hypotheses, predictors, moderators, mediators, outcomes, and findings. When information was missing, study authors were contacted via email.

During data extraction, H.B. created a detailed codebook (see supplementary materials) to ensure consistent coding. The codebook included key study details such as study design, type of digital media, sustainable food outcomes, digital attributes, and their effects. To ensure accuracy, two researchers (H.B. and L.G.) independently applied the codebook to all data. They measured agreement using Krippendorff’s Alpha, following Hayes and Krippendorff’s [21] guidelines. Agreement scores ranged from 0.549 to 0.916. Three variables scored below the acceptable threshold of 0.67: whether the paper described indirect effects of digital attributes (α = 0.549), whether it reported no effect of digital attributes (α = 0.633), and whether it described qualitative digital attributes influencing sustainable food outcomes (α = 0.610). These lower scores were mainly due to vague or inconsistent descriptions in the studies, which made coding more subjective. Disagreements were resolved by rereading and reinterpreting the relevant studies. Additionally, via an iterative consensus process involving three researchers (H.B., L.H., L.G.), all disagreements were reviewed in detail, strictly following the eligibility criteria and definitions in the codebook, until full agreement was reached. H.B performed data analysis by categorising, grouping, and visualising the extracted information through an iterative inductive process.

## Results

The results section is structured around the two main objectives of this review. First, we will outline the scope and volume of current evidence on the relationship between the digital food environment and consumers’ sustainable food outcomes. Second, we will map the attributes of the digital food environment that shape consumers’ sustainable food outcomes.

### Volume and scope of current evidence

**Fig. 2:**
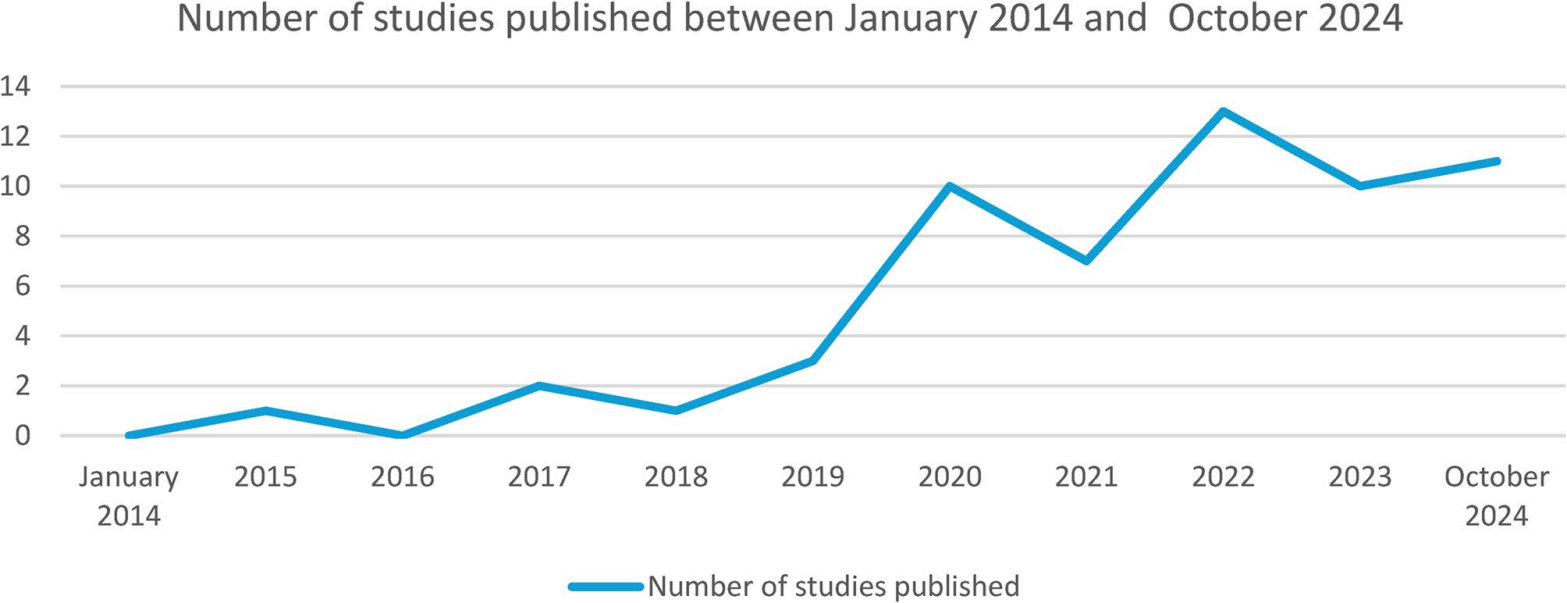
Number of studies published between January 2014 and October 2024

As evidenced in Fig. 2, publications focusing on the relationship between the digital food environment and sustainable food outcomes have been on an upward trend since 2014. This trend becomes especially pronounced from 2020 onwards and reaches its highest point so far in 2022.

**Fig. 3:**
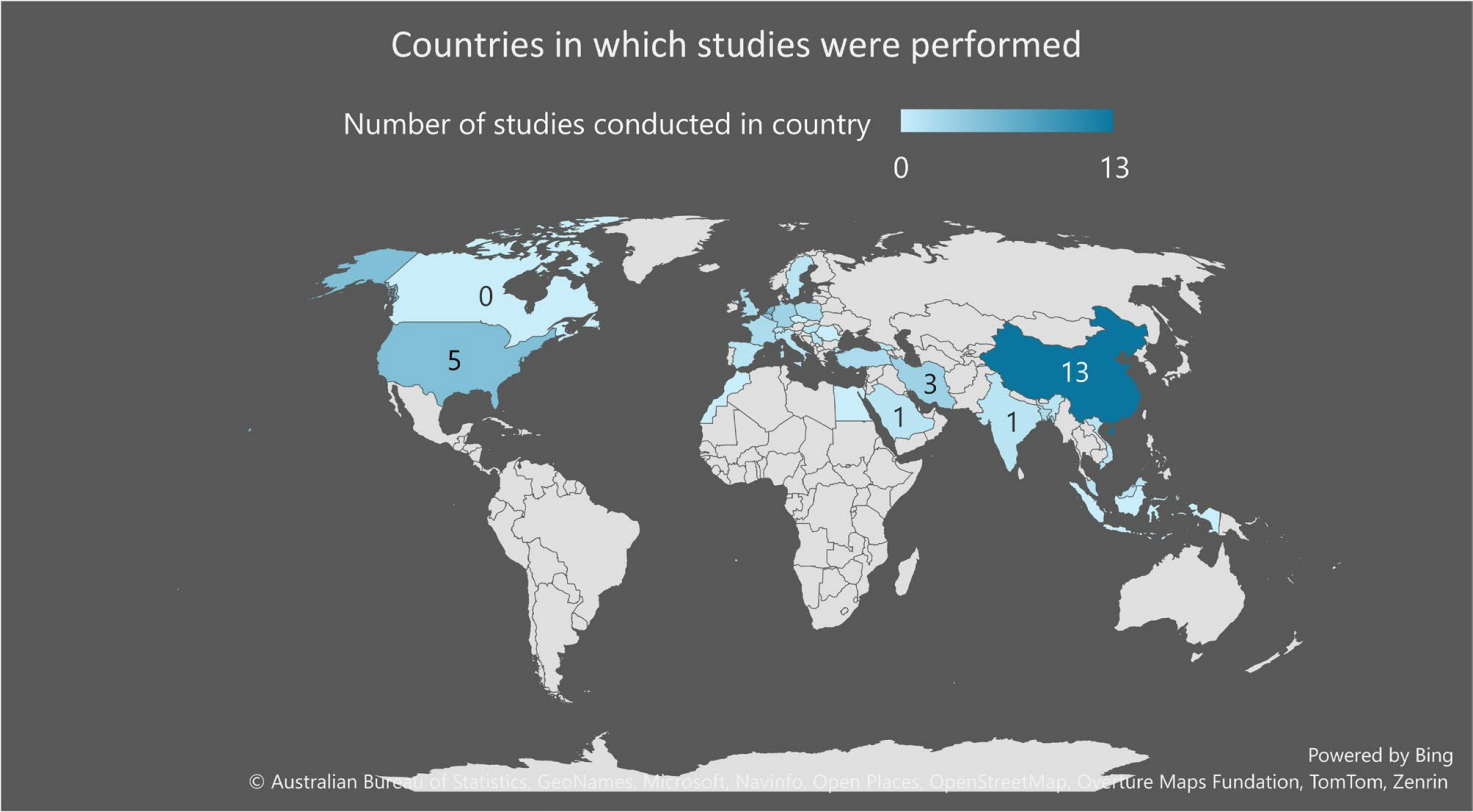
Countries in which studies were performed

As demonstrated in Fig. 3, the greater part of research in our sample (46.9% %; *n* = 30) was conducted in, what the World Bank Group [22] categorises as high-income countries, such as the Netherlands (*n* = 6). Additionally, about 39.1% of studies (*n* = 25) were performed in middle-income countries, such as China (*n* = 14). There were no low-income countries represented in the sample.

The majority of the identified studies were quantitative, including surveys (*n* = 29; 48.3%), experiments (*n* = 21; 35%), and observational studies (*n* = 2; 3.3%). Qualitative studies, such as interviews (*n* = 3; 5%) and focus groups (*n* = 2; 3.3%), were less common. Regarding socio-demographic characteristics, we found that, of those studies that reported the age of their samples (*n* = 52), the vast majority (98%, *n* = 51) focused on the general adult population. Only one paper specifically targeted children, and none focused exclusively on teenagers or older individuals. Additionally, an analysis of studies that reported their samples’ gender (*n* = 54) and educational level (*n* = 41) revealed that women and individuals with higher education (obtained or currently achieving a college degree) were more represented 1 compared to men and individuals with lower education. On average women made out 57% of the samples and people with higher education 73.8%. Less than half of the eligible studies (*n* = 25) provided some sort of metric (e.g., social class, monthly income, yearly income) to represent their sample’s socio-economic status. However, substantial variation in metrics across studies limited the ability to draw general conclusions.

### Attributes of the digital food environment

In total we identified 86 digital attributes across five types of digital media. The following section outlines the digital attributes identified in our sample, organised by different digital media, beginning with the most frequently cited (e-commerce) and ending with the least mentioned (blockchain). Table 2 gives an overview of the different attribute groups and their reoccurrence in the sample. A summary of all included studies, detailing their design, year of publication, medium examined, and the digital attributes mentioned, is provided in the supplementary materials.

**Table 2:** Overview of attributes groups and their reoccurrence in the sample

Attribute groups	Definition	Reoccurrence in studies included in the sample	Examples
E-commerce attributes	Digital attributes associated with platforms that are used to buy or sell food via the internet.	*N* = 33 (57.9% of total sample)	
E-commerce message strategy attributes	Digital attributes related to style and content of communication used on a food e-commerce platform.	*N* = 16 (28.1% of total sample)	e.g.,* feedback showing consumers how their food choices compare to others’ on an e-commerce platform* e.g.,* eco or nutri scores displayed on an e-commerce platform*
E-commerce platform characteristics attributes	Digital attributes pertaining to technical and structural features of food e-commerce platforms.	*N* = 20 (35.1% of total sample)	e.g.,* mediarichness of an e-commerce platform* e.g.,* usability of an e-commerce platform*
Social media attributes	Digital attributes related to social media platforms where people share content and connect with others	*N* = 18 (31.6% of total sample)	
Social media marketing attributes	Digital attributes related social media marketing, a specific form of online marketing that uses social media for promotion	*N* = 7 (12.3% of total sample)	e.g.,* interactive social media marketing content* e.g.,* customised social media marketing content*
Electronic word-of-mouth and user generated content attributes	Digital attributes associated with the online sharing of opinions, recommendations, and experiences (electronic word-of-mouth/e-Wom) and any social media content created by social media users (user generated content/UGC)	*N* = 8 (14% of total sample)	e.g.,* e-wom with peers* e.g.,* non-factual Youtube videos*
Influencer endorsement attributes	Digital attributes referring to social media influencers who promote a brand	*N* = 5 (8.8% of total sample)	e.g.,* congruence between a sustainable food product and the influencer promoting it* e.g.,* parasocial interactions between the audience and influencers promoting sustainable food*
Other media attributes	Digital attributes related to documentaries, apps that serve as sustainable food guides or blockchain technology	*N* = 9 (15.8% of total sample)	
Documentary attributes	Digital attributes associated with documentaries as factual audiovisual reports on particular subjects	*N* = 4 (7% of total sample)	e.g.,* documentaries with themes related to food sustainability*
Apps as sustainable food guide attributes	Digital attributes pertaining to apps that have the purpose to give consumers advise about sustainable food	*N* = 3 (5.3% of total sample)	e.g.,* speed of the app* e.g.,* customisation options in the app*
Blockchain attributes	Digital attributes relating to blockchain technology, a system that securely records data across multiple digital devices.	*N* = 2 (3.5% of total sample)	e.g.,* blockchain systems that tracks a food product’s journey from origin to consumer* e.g.,* blockchain systems that enable open information sharing about the product and its supply chain.*

### E-commerce

In total, 33 studies (57.9% of the sample) examined attributes related to e-commerce. We classified studies as e-commerce-related if they investigated digital attributes of platforms used to buy or sell food online (e.g., a supermarket’s online store or a food ordering app).

We divided e-commerce attributes into two categories: those relating to message strategies and those pertaining to platform characteristics. Message strategies refer to the style and content of communication used on food e-commerce platforms, while platform characteristics relate to the technical and structural features of these platforms.

#### Attributes of e-commerce platform characteristics

Sixteen studies (28.1% of the sample) explored e-commerce message strategy attributes, which refer to the content and style of communication used in food e-commerce settings.

Most of these studies (*n* = 12) focused on informative message attributes, which involve providing factual information about food products sold or bought on e-commerce platforms. Three qualitative studies found that participants felt having access to food product information, such the health benefits or environmental impact of food products, encouraged them to make more sustainable food choices. Additionally, inspirational food content, like meal plans or shopping lists featuring sustainable products, was viewed positively and helped motivate sustainable food behaviours [23–25]. Two quantitative studies showed that when participants were shown scores that reflected both the nutritional quality and the environmental impact of food products (nutri and eco-scores), it helped them make more sustainable food choices [26, 27]. Similarly, six quantitative studies found that sharing food sustainability information, such as the environmental impact of food products, improved consumers’ attitudes towards sustainable foods and increased their intention to buy them [28–34].

Four studies examined normative message strategy attributes, which involve communication that appeals to social norms influencing sustainable food choices. For instance, Berger [35] found that gamified feedback, showing consumers how their food choices compared to others’, encouraged more sustainable decisions. Similarly, sharing information about the sustainability of other consumers’ food purchases led to increased sustainable buying [36]. Personalised comparisons also boosted sustainable choices, but only when combined with recommendation agents (i.e., software suggesting healthier or more sustainable food options) and nutritional or eco-scores [37]. However, one study found that combining nudges, such as prominently placing sustainable foods, emphasising their appeal, and highlighting others’ sustainable food choices, had no significant impact on purchasing behaviour [38].

#### Attributes of e-commerce platform characteristics

We identified twenty studies (35.1% of the sample) that discussed e-commerce platform characteristics influencing sustainable food outcomes. Platform characteristics refer to the technical and structural features of food e-commerce environments. Across three qualitative studies, consumers indicated that design features, such as tools that visualise food shopping behaviour or allow data corrections (e.g., updating a delivery address), facilitated the adoption of more sustainable food practices [25, 39, 40]. Similarly, five quantitative studies found that certain platform features were linked to more positive attitudes towards sustainable food [41, 42]. For example, media-rich platforms, those that use multiple types of content and provide instant feedback, were linked to better consumer perceptions of sustainable food [43]. Adding features like a comment function also increased purchase intentions [44]. However, simply offering consistent and accurate access to data (e.g., delivery address, correcting order details) on e-commerce platforms did not lead to better sustainable food outcomes [31].

We identified seven studies that mentioned e-commerce platform attributes related to convenience. Convenient e-commerce platform attributes make shopping easier, faster, and more accessible. In two qualitative studies, consumers emphasised convenience as a key factor driving sustainable food behaviour [24, 25]. According to participants, convenience features on e-commerce platforms included time-saving options like saved shopping lists for quick reordering, flexible home delivery, a wide product range, and simple search functions [24, 40, 45]. The importance of convenience was also supported by three quantitative studies, which found that platform usability, ease of browsing, and product accessibility all contributed to more positive attitudes towards sustainable food and the platforms selling these products [31, 41, 46].

Furthermore, three quantitative studies examined e-commerce platform attributes related to overall platform quality. Two studies found that higher system quality improved consumer attitudes and purchasing behaviour in relation to sustainable food [47, 48]. Similarly, service quality, the accuracy and usefulness of information provided, and the effectiveness of the platform’s evaluation system were all linked to more favourable consumer attitudes towards sustainable food products [41, 48].

Six studies examined e-commerce platform attributes related to logistics. One quantitative study reported that effective e-commerce logistics can increase purchase intention of sustainable food [42]. Logistics was also featured as a recurring theme in three qualitative studies, where consumers noted that the inability to physically inspect food, limited geographical availability of an e-commerce platform, no-return policies, and inflexible pick-up times, hindered sustainable food behaviours [24, 39, 40]. Conversely, fast delivery was identified in one qualitative study as a driver of sustainable food choices [45]. However, while delivery speed emerged as a motivator in qualitative research, its influence on sustainable food attitudes varied in a quantitative study [48].

Three quantitative studies examined social commerce attributes that can influence sustainable food outcomes. Social commerce is a form of e-commerce in which transactions take place directly on social media platforms [49]. Examples of social commerce include caterers taking orders via WhatsApp Business or bakeries selling cakes through Facebook Marketplace. Social commerce attributes that can support sustainable food outcomes include: influencer endorsements (e.g., influencers promoting organic snacks), interactivity (e.g., Instagram polls about sustainable food), personalised recommendations (e.g., sustainable food suggestions based on previous social media activity), referrals (e.g., an invitation from a friend to follow a sustainable food page), feedback (e.g., customer ratings of sustainable restaurants), navigation design (e.g., filters to find sustainable products), visual appeal (e.g., attractive layouts), and informative value (e.g., details about a food product’s origin and environmental impact) [50–52]. However, the relationship between these features and sustainable food outcomes is not always consistent; for instance, reviews and ratings did not reliably affect sustainable food choices.

Three quantitative studies examined e-commerce live broadcasting attributes associated with sustainable food outcomes. E-commerce live broadcasting involves livestreams where food vendors or professional presenters showcase and sell products directly to online viewers [53]. E-commerce live broadcast platform characteristics linked to positive sustainable food behaviours and perceptions include: visibility (e.g., showing sustainable products during the livestream), authenticity (e.g., presenting genuine content), interactivity (e.g., allowing viewers to engage with the host), entertainment (e.g., making the livestream enjoyable), actively selling sustainable products during the stream, high-quality content, and the presence of supporting services [53, 54]. However, authenticity on its own was not found to significantly influence consumers’ attitudes towards sustainable food [55].

### Social media

Eighteen studies (31.6% of the total sample) examined social media attributes. Social media refers to online platforms where users create, share, and interact with content and each other. Social media attributes were grouped into three themes: social media marketing attributes, electronic word-of-mouth (e-Wom) and user-generated content (UGC) attributes, and influencer endorsement attributes. Social media marketing is defined here as a form of online marketing that uses social media platforms for promotional purposes. E-Wom refers to the online sharing of opinions, recommendations, and experiences, while user-generated content encompasses any material created by social media users. Influencer endorsement describes instances where social media influencers promote a brand.

#### Social media marketing

Social media marketing appears to play an important role in shaping sustainable food outcomes. We identified seven studies that examined social media marketing attributes. Four quantitative studies found that social media marketing was associated with increased sustainable food purchase and more positive perceptions of sustainable food [41, 56, 57]. These effects were particularly evident when the content was informative and interactive. In contrast, customised social media marketing content showed no measurable impact on sustainable food outcomes [58].

Findings from three qualitative studies also supported the influence of social media marketing. Consumers described it as a key driver of their sustainable food purchases, viewing such content as a valuable way to build trust in sustainable products [59]. Two studies noted that trust was strengthened when social media marketing messages emphasised transparent production standards (e.g., low-carbon farming methods) and the legitimacy of the supply chain (e.g., verified suppliers, traceable product origins) [60, 61].

#### User-generated content and electronic word-of-mouth

Eight studies in our sample highlighted user-generated content (UGC) and electronic word-of-mouth (e-Wom) as important factors influencing sustainable food outcomes. E-Wom refers to the online sharing of opinions, recommendations, and experiences about sustainable food. Consumers who engaged in e-WOM tended to hold more positive attitudes towards sustainable food, although this did not necessarily translate into higher purchase intentions [58, 62]. Interestingly, one study found that consumers perceived greater risks when engaging in e-Wom with experts rather than with peers or anonymous users [63]. Positive e-WOM comments, as well as the technical design of the e-WOM platform, were also found to improve perceptions of sustainable food [64, 65].

Two qualitative studies identified UGC, customer-created content shared on social media, as a driver of sustainable food behaviour [24, 66]. However, quantitative findings were more mixed; for example, Koswatta et al. [67] found that non-factual YouTube videos had inconsistent effects on perceptions of sustainable food.

#### Influencer endorsements

Influencer endorsement was highlighted in five studies as an important factor influencing sustainable food outcomes. For example, the qualitative study of Choudhary et al. [66] found that consumers viewed online influencers as role models who helped shape their adoption of sustainable food behaviours. Quantitative research further supported the impact of influencer endorsement on sustainable food choices [68]. One study showed that influencers’ self-disclosure (i.e., sharing their own sustainable food experiences) increased positive attitudes towards sustainable food [69]. Additionally, factors such as parasocial interactions (i.e., the sense of a personal connection consumers feel with influencers), active audience participation, the perceived benevolence of influencers, and how well the influencer’s image matched the product, all contributed to stronger intentions to purchase sustainable food products [70, 71].

### Other media

The following section outlines three less frequently examined types of digital media: apps, documentaries, and blockchain.

#### Apps as sustainable food guides

Two quantitative studies found that apps designed as sustainable food guides positively influenced sustainable food outcomes. Key app features such as fast performance, relevant content, customisation options, and the availability of sufficient information, all improved consumers’ perceptions of these apps [72]. Additionally, providing basic eco-rankings of products within an app helped reduce consumers’ uncertainty when making choices and encouraged more sustainable food decisions [73]. However, more detailed eco-rankings only increased sustainable choices when the information was consistent and consumers did not have to make difficult trade-offs. In a qualitative study by Vos et al. [24], consumers suggested that adding a filter function to sustainable food apps could further enhance their usefulness.

#### Documentaries

Three quantitative studies in our sample found that a documentary with a sustainability theme could increase sustainable food outcomes [74–76] while reducing unsustainable food outcomes [77].

#### Blockchain

Two quantitative studies investigated blockchain attributes. A blockchain-based food traceability system, a method that tracks a product’s journey from its origin to the consumer, was associated with positive sustainable food outcomes, including increased trust and purchase intention [78, 79]. Furthermore, both this traceability system and blockchain-enabled transparency, which involves the clear and open sharing of information about the product and its supply chain, were found to enhance consumers’ intention to buy sustainable products [78].

## Discussion

This scoping review aimed to map the existing evidence on attributes of the digital food environment that shape consumers’ sustainable food outcomes. We identified 86 digital attributes across five types of digital media.

E-commerce emerged as the most frequently discussed digital medium, mentioned in 33 studies (57.9% of total sample). In terms of e-commerce messaging strategies (mentioned in 16 studies), informative attributes were found to be the most prevalent (cited in 12 studies). Three qualitative studies indicated that consumers regard information relating to sustainable food as a key driver of their sustainable food behaviour, a finding further supported by six quantitative studies. This aligns with previous research suggesting that food literacy, which encompasses knowledge, skills, attitudes, and practices related to food, plays a crucial role in shaping sustainable food outcomes [80]. Concerning e-commerce platform attributes, design elements were most frequently mentioned. Five qualitative studies highlighted features, such as visualisation and transparency, as drivers of consumers’ sustainable food behaviour. Similarly, three quantitative studies showed that design characteristics, like media richness, are positively related to sustainable food outcomes. The prominence of design attributes in our sample is unsurprising, as e-commerce design has long been recognised as crucial to consumers’ purchase decisions [81].

Social media was the second most frequently cited digital medium (cited in 18 studies, 31.6% of total sample), with most studies (*n* = 7) mentioning social media marketing attributes. Four quantitative studies highlighted the influence of social media marketing on sustainable food purchases. These findings were further supported by three qualitative studies, which identified social media marketing as a key factor in building trust. Previous research has shown the significant impact of social media marketing on sustainable behaviours [82, 83], and our findings suggest this effect may also extend to sustainable food.

Studies on e-commerce (*n* = 33) and social media (*n* = 18) attributes dominate our sample, aligning with previous reviews on the digital food environment that highlight these media as key areas of focus (e.g [9, 11]). However, despite the extensive research on e-commerce and social media, other digital media types, such as documentaries, blockchain, and apps designed as sustainable food guides, have been less explored. Only 15.8% of the studies in our sample focused on these media. As such, there is much potential for future research to investigate how these underexplored digital media relate to sustainable food outcomes.

Our sample was primarily quantitative and disproportionately focused on high- and middle-income countries, consistent with other reviews (e.g [11, 84]). Additionally, women and highly educated individuals were overrepresented, reflecting research that shows these groups are more likely to purchase sustainable food [85, 86]. In contrast, men, children, teenagers, older adults, and those with lower socio-economic status or education were underrepresented. As such, there is significant potential for future research to explore these underexamined groups.

### Limitations

Our search string was limited to English-language, peer-reviewed articles published between 2014 and October 2024. As a result, relevant studies published after October 2024 or in other languages may have been excluded. Furthermore, the search string was developed with the intention of providing a broad overview of attributes in the digital food environment that shape sustainable outcomes. Consequently, this review does not offer an in-depth analysis of individual attributes within the digital food environment. Additionally, some digital attribute codes demonstrated lower inter-rater reliability, largely due to vague or inconsistent reporting in the primary studies, which introduced subjectivity into coding. Finally, most studies in our sample focused on high- and middle-income countries, which limits their generalisability to low-income settings.

## Conclusion

This scoping review summarises existing research on how the digital food environment relates to consumers’ sustainable food outcomes (i.e. consumers' perceptions, attitudes, intentions, and behaviours related to sustainable food). Drawing on 57 studies, 86 distinct digital attributes were identified across five categories of digital media. E-commerce was the most frequently examined medium (57.9% of studies), with attributes primarily emphasising informative messaging and platform design features. Social media was the second most cited (28.1%), with attributes largely focused on social media marketing strategies. The review showed that current studies are predominantly conducted in high income countries (46.9%) and tend to focus on adult, female, and highly educated populations. Alternative forms of digital media (e.g., documentaries) and underrepresented groups (e.g., individuals on low incomes) remain significantly underexplored. Future research should explore a wider array of media channels and population groups to deepen insights into the relationship between digital attributes and sustainable food outcomes.

## Supplementary Information

Supplementary Material 1.

Supplementary Material 2.

Supplementary Material 3.

Supplementary Material 4.

## Data Availability

The datasets generated and/or analysed during the current study are available in the Open Science Framework repositor, OSF | A Scoping Review of Factors in the Digital Food Environment that Influence Consumers’ Sustainability Perceptions and Behaviours. The search strings used for searching relevant literature are included within the article additional files.

## Footnotes

1 This assessment was conducted by extracting descriptive information on education and gender from all included studies where such data were available. We then analysed these data to identify which groups were most prominently represented on average across the samples. This approach was based on the reported participant characteristics within each study rather than independently comparing them to external population benchmarks.

## References

[1] Crippa M, Solazzo E, Guizzardi D, Monforti-Ferrario F, Tubiello FN, Leip A. Food systems are responsible for a third of global anthropogenic GHG emissions. Nat Food. 2021;2(3):198–209. 37117443 10.1038/s43016-021-00225-9

[2] FAO. The future of food and agriculture: Drivers and triggers for transformation. 2022 [Cited 2024 Dec 11]. Available from: https://openknowledge.fao.org/items/594ccd69-8582-4dc7-9832-18bfdf44cedd

[3] FAO. The State of Food Security and Nutrition in the World 2019. Safeguarding against economic slowdowns and downturns. The State of Food Security and Nutrition. 1st ed. Bloomfield: United Nations; 2019. p. 1. in the World Series).

[4] Lindgren E, Harris F, Dangour AD, Gasparatos A, Hiramatsu M, Javadi F, et al. Sustainable food systems—a health perspective. Sustain Sci. 2018;13(6):1505–17. 30546484 10.1007/s11625-018-0586-x PMC6267166

[5] European Commission, COMMUNICATION FROM THE COMMISSION TO THE EUROPEAN PARLIAMENT, THE EUROPEAN COUNCIL, THE COUNCIL, THE EUROPEAN ECONOMIC AND SOCIAL COMMITTEE AND THE COMMITTEE OF THE REGIONS The European Green Deal. 2019. Accessed August 18, 2025. Available from: https://eur-lex.europa.eu/legal-content/EN/TXT/?uri=CELEX%3A52019DC0640.

[6] UN. Transforming our world : the 2030 Agenda for Sustainable Development. 2015. Available from: https://www.refworld.org/legal/resolution/unga/2015/en/111816.

[7] Marvin HJP, Bouzembrak Y, van der Fels-Klerx HJ, Kempenaar C, Veerkamp R, Chauhan A, et al. Digitalisation and artificial intelligence for sustainable food systems. Trends Food Sci Technol. 2022;120:344–8.

[8] Granheim SI, Opheim E, Terragni L, Torheim LE, Thurston M. Mapping the digital food environment: a scoping review protocol. BMJ Open. 2020;10(4):e036241. 32327482 10.1136/bmjopen-2019-036241 PMC7204828

[9] Granheim SI, Løvhaug AL, Terragni L, Torheim LE, Thurston M. Mapping the digital food environment: a systematic scoping review. Obes Rev. 2022;23(1):e13356. 34519396 10.1111/obr.13356

[10] Granheim S. The digital food environment. Nutrition. 2019;44:33–7.

[11] Bennett R, Keeble M, Zorbas C, Sacks G, Driessen C, Grigsby-Duffy L, et al. The potential influence of the digital food retail environment on health: a systematic scoping review of the literature. Obes Rev. 2024;25(3):e13671. 38104965 10.1111/obr.13671

[12] Wyse R, Jackson JK, Delaney T, Grady A, Stacey F, Wolfenden L, et al. The effectiveness of interventions delivered using digital food environments to encourage healthy food choices: A systematic review and Meta-Analysis. Nutrients. 2021;13(7):2255. 34208869 10.3390/nu13072255 PMC8308236

[13] WHO. Digital food environments: factsheet. 2021 [Cited 2024 Dec 3]. Available from: https://www.who.int/europe/publications/i/item/WHO-EURO-2021-2755-42513-59052.

[14] Food and Agriculture Organization of the United Nations (FAO). International scientific symposium: biodiversity and sustainable diets – united against hunger; 2010 Nov 3–5; Rome, Italy. Rome: FAO; 2012.

[15] Boen H, Glenisson L, Hallez L, Smits T. A Scoping Review Protocol for Examining Factors in the Digital Food Environment that Influence Consumers’ Sustainability Perceptions and Behaviours. 2024 Nov 20 [Cited 2024 Dec 3]; Available from: https://osf.io/sx5dk

[16] Tricco AC, Lillie E, Zarin W, O’Brien KK, Colquhoun H, Levac D, et al. PRISMA extension for scoping reviews (PRISMA-ScR): checklist and explanation. Ann Intern Med. 2018;169(7):467–73. 30178033 10.7326/M18-0850

[17] Kristia K, Kovács S, László E. Food delivery platform and food waste: deciphering the role of promotions, knowledge, and subjective norms among Indonesian generation Z. Cleaner and Responsible Consumption. 2023;11:100152.

[18] Gerasimova K. Debates on genetically modified crops in the context of sustainable development. Sci Eng Ethics. 2016;22(2):525–47. 26062746 10.1007/s11948-015-9656-y

[19] Glenisson L, Hallez L, Smits T. Thought for food: A systematic review of how psychological state factors affect sustainable food outcomes. Sustain Prod Consum. 2025;57:277–91.

[20] Forbes C, Greenwood H, Carter M, Clark J. Automation of duplicate record detection for systematic reviews: deduplicator. Syst Rev. 2024; 13(1):206. 10.1186/s13643-024-02619-9 PMC11295717 39095913

[21] Marzi G, Balzano M, Marchiori D. K-Alpha Calculator–Krippendorff’s Alpha Calculator: A user-friendly tool for computing Krippendorff’s Alpha inter-rater reliability coefficient. MethodsX. 2024;12:102545. 39669968 10.1016/j.mex.2023.102545 PMC11636850

[22] World Bank. The world by income and region [Internet]. Washington (DC): The World Bank; [cited 2025 Aug 18]. Available from: https://datatopics.worldbank.org/world-development-indicators/the-world-by-income-and-region.html.

[23] Smit ES, Meijers MHC, van der Laan LN. Using virtual reality to stimulate healthy and environmentally friendly food consumption among children: an interview study. Int J Environ Res Public Health. 2021;18(3):1088. 33530495 10.3390/ijerph18031088 PMC7908483

[24] Vos M, Deforche B, Van Kerckhove A, Michels N, Geuens M, Van Lippevelde W. Intervention strategies to promote healthy and sustainable food choices among parents with lower and higher socioeconomic status. BMC Public Health. 2022;22(1):2378. 36536355 10.1186/s12889-022-14817-y PMC9761028

[25] Kaiser M, Ryan-Simkins K, Dionne J, Pence E. Connecting small-scale producers and consumers: exploring the feasibility of online food hubs in low-income communities. J Agric Food Syst Community Dev. 2020;9(3):179–96.

[26] Kanay A, Hilton D, Charalambides L, Corrégé JB, Inaudi E, Waroquier L, et al. Making the carbon basket count: goal setting promotes sustainable consumption in a simulated online supermarket. J Econ Psychol. 2021;83:102348.

[27] De Bauw M, Matthys C, Poppe V, Franssens S, Vranken L. A combined Nutri-Score and ‘Eco-Score’ approach for more nutritious and more environmentally friendly food choices? Evidence from a consumer experiment in Belgium. Food Qual Prefer. 2021;93:104276.

[28] Andreani G, Sogari G, Wongprawmas R, Menozzi D, Mora C. Indulgent or informative logos? Effects on university students’ intention to purchase healthy and sustainable food. Int J Gastron Food Sci. 2023;33:100774.

[29] Danner H, Thøgersen J. Does online chatter matter for consumer behaviour? A priming experiment on organic food. Int J Consum Stud. 2022;46(3):850–69.

[30] Meijers MHC, Smit ES, de Wildt K, Karvonen SG, van der Plas D, van der Laan LN. Stimulating sustainable food choices using virtual reality: taking an environmental vs health communication perspective on enhancing response efficacy beliefs. Environ Commun. 2022;16(1):1–22.

[31] Pahari S, Ghosal I, Prasad B, Dildar SM. Which determinants impact consumer purchase behavior toward online purchasing of organic food products? Prabandhan Indian J Manag. 2023;16(1):25–41.

[32] Potter C, Pechey R, Clark M, Frie K, Bateman PA, Cook B, et al. Effects of environmental impact labels on the sustainability of food purchases: A randomised controlled trial in an experimental online supermarket. PLoS ONE. 2024;3(9):e0309386. 10.1371/journal.pone.0309386 PMC11371233 39226274

[33] van der Waal NE, Folkvord F, Azrout R, Meppelink CS. Can product information steer towards sustainable and healthy food choices? A pilot study in an online supermarket. Int J Environ Res Public Health. 2022;19(3):1107. 35162129 10.3390/ijerph19031107 PMC8834331

[34] Wu Y, Fu S, Long R, Islam Md S, Huang E. Do green information transparency and exposure always boost online sales of organic food? An evidence from China. Electron Commer Res Appl. 2024;65:101400.

[35] Berger V. Social norm-based gamification to promote eco-friendly food choice. J Consum Market. 2019;36(5):666–76.

[36] Demarque C, Charalambides L, Hilton DJ, Waroquier L. Nudging sustainable consumption: The use of descriptive norms to promote a minority behavior in a realistic online shopping environment. J Environ Psychol. 2015;43:166–74.

[37] De Bauw M, De La Revilla LS, Poppe V, Matthys C, Vranken L. Digital nudges to stimulate healthy and pro-environmental food choices in e-groceries. Appetite. 2022;172:105971. 35181380 10.1016/j.appet.2022.105971

[38] van der Vliet N, Stuber JM, Raghoebar S, Roordink E, van der Swaluw K. Nudging plant-based alternatives to meat and dairy in a real-life online supermarket: a randomized controlled trial. Appetite. 2024;196:107278. 38373537 10.1016/j.appet.2024.107278

[39] Katzeff C, Milestad R, Zapico JL, Bohné U. Encouraging organic food consumption through visualization of personal shopping data. Sustainability. 2020;12(9):3599.

[40] Barska A, Wojciechowska-Solis J. E-consumers and local food products: a perspective for developing online shopping for local goods in Poland. Sustainability. 2020;12(12):4958.

[41] Kocic M, Sapic S, Sofronijevic K. The influence of website quality on cognitive and affective attitudes towards organic food. Ekon Horizonti. 2022;24(3):299–312.

[42] Lin J, Li T, Guo J. Factors influencing consumers’ continuous purchase intention on fresh food e-commerce platforms: an organic foods-centric empirical investigation. Electron Commerce Res Appl. 2021;50:101103.

[43] Yue L, Liu Y, Wei X. Influence of online product presentation on consumers’ trust in organic food: a mediated moderation model. Br Food J. 2017;119(12):2724–39.

[44] Zhang J, Rudnák I. Evaluation of the factors affecting consumers’, purchases of fresh food online from China and Hungary. Int J EBusiness EGovernment Stud. 2023;15(2):212–30.

[45] Zámková M, Prokop M, Šimpach O, Smutka L, Tomšík K, Vavrečka V. Czech consumers' preference for organic products in online grocery stores during the COVID-19 pandemic. Int J Environ Res Public Health. 2022;19(20):13316. 10.3390/ijerph192013316 PMC9603500 36293910

[46] Robina-Ramírez R, Chamorro-Mera A, Moreno-Luna L. Organic and online attributes for buying and selling agricultural products in the e-marketplace in Spain. Electron Commer Res Appl. 2020;42:100992.

[47] Prince SA, Wahid IS, Islam SA. Consumption Value and Organic Food Purchasing Behavior of Online Consumers. J Risk Anal Crisis Response. 2024 June 30 [Cited 2024 Nov 21];14(2). Available from: https://jracr.com/index.php/jracr/article/view/468

[48] Qi X, Lv X, Li Z, Yang C, Li H, Ploeger A. Factors influencing young adults’ organic food purchase intention on fresh food e-commerce platforms. Br Food J. 2024;126(12):4277–303.

[49] Esmaeili L, Hashemi G. SA. A systematic review on social commerce. J Strateg Mark. 2019;27(4):317–55.

[50] Lin J, Guo J, Turel O, Liu S. Purchasing organic food with social commerce: An integrated food-technology consumption values perspective. Int J Inf Manag. 2020 [Cited 2024 Nov 21];51(C). Available from: https://ideas.repec.org//a/eee/ininma/v51y2020ics0268401219304979.html

[51] Poureisa A, Aziz YA, Ng SI. Swipe to sustain: exploring consumer behaviors in organic food purchasing via Instagram social commerce. Sustainability. 2024;16(6):2338.

[52] Tariq A, Wang C, Tanveer Y, Akram U, Akram Z. Organic food consumerism through social commerce in China. Asia Pac J Mark Logist. 2019;31(1):202–22.

[53] Guo H, Sun X, Pan C, Xu S, Yan N. The sustainability of fresh agricultural produce live broadcast development: influence on consumer purchase intentions based on live broadcast characteristics. Sustainability. 2022;14(12):7159.

[54] Wang Y, Fang L, Pan J. The antecedents of customer satisfaction in the live-streaming commerce of green agricultural products. PLoS ONE. 2024;19(7):e0305527. 38995893 10.1371/journal.pone.0305527 PMC11244768

[55] Song Z, Liu C, Shi R. How do fresh live broadcast impact consumers’ purchase intention? Based on the SOR theory. Sustainability. 2022;14(21):14382.

[56] Armutcu B, Ramadani V, Zeqiri J, Dana LP. The role of social media in consumers’ intentions to buy green food: evidence from Türkiye. Br Food J. 2024;126(5):1923–40.

[57] Kim EJ, Kim EL, Kim M, Tang J. The matching effect of local food and color on ethical dining behaviors: the roles of credibility and green image. Int J Contemp Hosp Manage. 2023;36(5):1557–76.

[58] Lian S, Liew C, Subramaniam M, Ahamad S. The effect of social media marketing on consumers’ purchase intention of organic food: the role of perceived value, trust and social identity. Int J Electron Mark Retail. 2024;15:519–40.

[59] Wu Y, Takács-György K. Comparison of consuming habits on organic food—is it the same? Hungary versus China. Sustainability. 2022;14(13):7800.

[60] Tashakkori E, Sobhanifard Y, Jamshidi R, Sadeghi ME. A modeling for enhancing consumer trust in organic food through authentic content in social networks. Int J Innov Manag Econ Soc Sci. 2023;3(4):1–17.

[61] Sobhanifard Y. Hybrid modelling of the consumption of organic foods in Iran using exploratory factor analysis and an artificial neural network. Br Food J. 2018;120(1):44–58.

[62] Zayed MF, Gaber HR, El Essawi N. Examining the factors that affect consumers’ purchase intention of organic food products in a developing country. Sustainability. 2022;14(10):5868.

[63] Hilverda F, Kuttschreuter M, Giebels E. Social media mediated interaction with peers, experts and anonymous authors: conversation partner and message framing effects on risk perception and sense-making of organic food. Food Qual Prefer. 2017;56:107–18.

[64] Hilverda F, Kuttschreuter M, Giebels E. The Effect of Online Social Proof Regarding Organic Food: Comments and Likes on Facebook. Front Commun [Internet]. 2018 Aug 3 [cited 2024 Nov 21];3. Available from: https://www.frontiersin.org/journals/communication/articles/10.3389/fcomm.2018.00030/full

[65] You JJ, Jong D, Wiangin U, Consumers. ’ Purchase Intention of Organic Food via Social Media: The Perspectives of Task-Technology Fit and Post-acceptance Model. Front Psychol. 2020 Nov 5 [Cited 2024 Nov 21];11. Available from: https://www.frontiersin.org/journals/psychology/articles/10.3389/fpsyg.2020.579274/full 10.3389/fpsyg.2020.579274 PMC7674305 33224070

[66] Choudhary S, Nayak R, Kumari S, Choudhury H. Analysing acculturation to sustainable food consumption behaviour in the social media through the lens of information diffusion. Technol Forecast Soc Change. 2019;145:481–92.

[67] Koswatta TJ, Wingenbach G, Leggette HR. Effect of information on public perception of organic foods: a case study. Br Food J. 2023;125(7):2514–39.

[68] Kalam A, Monirul Islam SM, Akterujjaman SM. Momentum for organic food purchase intention and actual adoption- moderating effects of social media influencer and celebrity endorser. Food Qual Prefer. 2025;122:105307.

[69] Wu Y, Yang S, Liu D. The effect of social media influencer marketing on sustainable food purchase: Perspectives from multi-group SEM and ANN analysis. J Clean Prod. 2023;416:137890.

[70] Xu Z, Islam T, Liang X, Akhtar N, Shahzad M. I’m like you, and i like what you like’ sustainable food purchase influenced by vloggers: a moderated serial-mediation model. J Retailing Consumer Serv. 2021;63:102737.

[71] Al-Harbi AI, Badawi NS. Can opinion leaders through Instagram influence organic food purchase behaviour in Saudi Arabia? J Islam Mark. 2021;13(6):1312-33. 10.1108/JIMA-01-2020-0025.

[72] Matin A, Khoshtaria T, Todua N, Bareja-Wawryszuk O, Pajewski T, Todua N. Determinants of Green Smartphone Application Adoption for Sustainable Food Consumption Among University Students. Int J Mark Commun New Media. 2024 Jan 19 [Cited 2024 Nov 21];11(21). Available from: http://u3isjournal.isvouga.pt/index.php/ijmcnm/article/view/794

[73] Weber A. Mobile apps as a sustainable shopping guide: the effect of eco-score rankings on sustainable food choice. Appetite. 2021;167:105616. 34358589 10.1016/j.appet.2021.105616

[74] Pabian S, Hudders L, Poels K, Stoffelen F, De Backer CJS. Ninety Minutes to Reduce One’s Intention to Eat Meat: A Preliminary Experimental Investigation on the Effect of Watching the Cowspiracy Documentary on Intention to Reduce Meat Consumption. Front Commun. 2020 Aug 28 [Cited 2024 Nov 21];5. Available from: https://www.frontiersin.org/journals/communication/articles/10.3389/fcomm.2020.00069/full

[75] Ma J, Seenivasan S, Yan B. Media influences on consumption trends: effects of the film Food, Inc. on organic food sales in the U.S. Int J Res Mark. 2020;37(2):320–35.

[76] Bschaden A, Mandarano E, Stroebele-Benschop N. Effects of a documentary on consumer perception of the environmental impact of meat consumption. Br Food J. 2020;21(1):177–89.

[77] Herchenroeder L, Forestell CA, Bravo AJ. The effectiveness of animal welfare-, environmental-, and health-focused video appeals on implicit and explicit wanting of meat and intentions to reduce meat consumption. J Soc Psychol. 2023;163(3):394–407. 35670371 10.1080/00224545.2022.2081529

[78] Ho BD, Duong DC, Ngo VNT, Nguyen HM, Bui VT. How blockchain-enabled drivers stimulate consumers’ organic food purchase intention: an integrated framework of information systems success model within stimulus-organism-response theory in the context of Vietnam. Int J Human–Comput Interact. 2024;0(0):1–19.

[79] Tao Z, Chao J. The impact of a blockchain-based food traceability system on the online purchase intention of organic agricultural products. Innov Food Sci Emerg Technol. 2024;92:103598.

[80] Teng CC, Wang YC, Cheng YJ, Wang SN. Religious beliefs and food waste prevention practices: mechanisms of divine and environmental awareness. J Hosp Mark Manag. 2023;32(4):530-54.

[81] Modi N, Singh J. Understanding online consumer behavior at E-commerce portals using eye-gaze tracking. Int J Human-Comput Interact. 2023;39(4):721–42.

[82] Handoko W, Roziki AA, Ferdinand AT. The effect of green social media marketing on purchase decision: A systematic literature review. Res Horiz. 2024;4(4):361–8.

[83] Bryła P, Chatterjee S, Ciabiada-Bryła B. The impact of social media marketing on consumer engagement in sustainable consumption: a systematic literature review. Int J Environ Res Public Health. 2022;19(24):16637. 36554529 10.3390/ijerph192416637 PMC9779249

[84] Lanham AR, van der Pols JC. Toward sustainable diets: interventions and perceptions among adolescents: a scoping review. Nutr Rev. 2025;83(2):e694-e710. 10.1093/nutrit/nuae052 PMC11723159 38809755

[85] Mohr M, Schlich M. Socio-demographic basic factors of German customers as predictors for sustainable consumerism regarding foodstuffs and meat products. Int J Consum Stud. 2016;40(2):158–67.

[86] Dominici A, Boncinelli F, Gerini F, Marone E. Determinants of online food purchasing: the impact of socio-demographic and situational factors. J Retail Consum Serv. 2021;60:102473.

