# Supplementary material for: Byte into sustainability: a scoping review of digital food environment attributes that shape consumers’ sustainability perceptions, attitudes, intentions, and behaviours
Source: Int J Behav Nutr Phys Act. 2025 Oct 27;22:132. doi: 10.1186/s12966-025-01832-6 (PMC12560354; doi:10.1186/s12966-025-01832-6)
Supplement: Supplementary file 1 — Supplementary Material 1. [file 12966_2025_1832_MOESM1_ESM.docx]

| **Values** | **Notes** | **Examples** |
| --- | --- | --- |
| Survey (1) Interview (2) Experiment (3) Focus group (4) Mixed (5) specify in notes which designs were mixed Observational study (6) | **Qualitative pretests do not count as a full-fledged elements of a mixed design. Only if both designs are equally discussed in the paper, it can be coded as mixed.** |  |
| Ecommerce (1) Social Media (2)  Apps (3)  Documentary (4)  Social Commerce (5)  Live Broadcast (6)  Blockchain (7) Other (8) specify… | **What is the general digital context of the study?  Ecommerce:** Studies about online supermarkets or apps you can use to buy sustainable food (bv: online supermarket, food ordering apps)  **Social media:** Studies about social media content or social media characteristics which **do NOT** explicitly elaborate on commerce through social media. Studies about social media used for ecommerce are categorised as social commerce.  **Apps as sustainable food guides:** Studies about apps which stimulate sustainable food consumption/purchase which **do NOT elaborate** on commerce via apps . Studies focused on apps that are meant for commerce are categorised as ecommerce.  **Documentary:** Studies about the impact of video/film/documentary on sustainable food outcomes (e.g., the impact of a Netflix documentary on the sales of organic eggs). Studies about youtube can be coded as social media.  **Social commerce**: Studies about social media explicitly used for ecommerce of sustainable food.  **Live broadcast**: Studies about broadcasting related to sustainable food outcomes  **Blockchain:** Studies about blockchain technology related to sustainable food outcomes |  |
| Yes (1) No (2) Not applicable (3) | **Digital factors**: characteristics of the digital medium of which the effect on sustainability outcomes was statistically measured (e.g., authentiticy of an influencer). It does not include consumers' perceptions about those characteristics (e.g., perceptions about authenticity of an influencer)  **Yes:** The study's results proved that one or more digital factors have a direct effect on a sustainable food outcome **No**: The study results did not prove that that one or more digital factors have a direct effect on a sustainable food outcome **Not applicable:**  The study did not investigate digital factors, but rather digital drivers/inhibitors.  **Specify in notes which digital factors you could extract** | **Example of a direct effect:**   Website quality (digital factor) has an impact on purchase intention of organic food  Influencer authentiticy (digital factor) has an impact on purchase intention of local food |
| Yes (1) No (2) Not applicable (3) | **Digital factors**: characteristics of the digital medium of which the effect on outcomes was statistically measured. It does not include consumers' perceptions about those characteristics.  **Yes:** The study's results proved that one or more digital factors have an indirect effect on a sustainable food outcome **No**: The study results did not prove that that one or more digital factors have an indirect effect on a sustainable food outcome **Not applicable:**  The study did not investigate digital factors, but rather digital drivers/inhibitors.  Interaction effects are coded as indirect effects.   **Specify in notes which digital factors you could extract and if they were a moderator, mediator or independent variable within the indirect effect.** | **Example of an indirect effect:**  **Digital factor as moderator**: Purchase intention has an impact on purchase behaviour moderated by exposure to ecolabels (digital factor)  **Digital factor as mediator**: Purchase intention has an impact on purchase behaviour via ecofriendly feedback (digital factor)  **Digital factor as independent variable:** Authenticity of livebroadcast (digital factor) has an impact on purchase intention via task characteristics |
| Yes (1) No (2) Not applicable (3) | **Digital factors:** characteristics of the digital medium of which the effect on outcomes was statistically measured. It does not include consumers' perceptions about those characteristics.  **Yes**: The study's results mention that one or more digital factors are part of an unsignificant effect. **No:** The study's results mention that all digital factors effects have reached statistical significance. **Not applicable:** The study did not investigate digital factors, but rather digital drivers/inhibitors.  **Specify in notes which digital factors you could extract.** | **Example of no effect:**  Media richness(digital factor) has no effect on perceived credibility of a sustainable message |
| Yes (1) Not applicable (2) | **Digital drivers/inhibitors**: characteristics of a digital medium that were not tested for effect size. It does not include consumers' perceptions about those characteristics.  **Yes**: The study mentioned digital drivers/inhibitors that can influence sustainable food outcomes **No:** The study did not mention digital drivers/inhibitors that can influence sustainable food outcomes **Not applicable:** The study did not investigate digital drivers/inhibitors, but rather digital factors  **Specify in notes which digital factors you could extract and if they are mentioned as a driver (D), as an inhibitor (I) or both (D/I) of outcomes.** | **Example of a driver:** Participants explained that ease of searching in the app (digital driver) motivated them to buy organic food  **Example of an inhibitor**: Some consumers stated that ecorankings (digital inhibitor) hindered their purchase intention   **Example of a driver and inhibitor:** Most young people said that using social media (digital driver and inhibitor) as inspiration for sustainable cooking helped them decrease their meat intake, but also made them experience decision uncertainty |
| Perceptions (1) Values (2) Feelings (3) Attitudes (4)  Cognitions (5)  Motivation (6) Intention (7)  Behaviour (8) Other (9), specify | **Specify in notes which specific outcomes you could extract.** |  |
