# Supplementary material for: Byte into sustainability: a scoping review of digital food environment attributes that shape consumers’ sustainability perceptions, attitudes, intentions, and behaviours
Source: Int J Behav Nutr Phys Act. 2025 Oct 27;22:132. doi: 10.1186/s12966-025-01832-6 (PMC12560354; doi:10.1186/s12966-025-01832-6)
Supplement: Supplementary file 2 — Supplementary Material 2. [file 12966_2025_1832_MOESM2_ESM.docx]

2014-now

WoS:

(((((TS=(Human OR Consumer* OR Adult* OR People OR Senior* OR Man OR Men OR Woman OR Women OR Child* OR Teen* OR Student* OR Adolescen* OR Young OR Boy* OR Girl* OR Gen X OR Gen Y OR Gen Z OR Vulnerable OR low-income OR Marginalized OR Disadvantaged OR Elderly OR immigrant* OR refugee* OR ethnic OR minority OR Indigenous OR unemployed OR customer* OR individual OR family OR household OR father* OR mother* OR parent*))

AND TS=(Character* OR Emotional state OR Personal conviction*OR Political preference* OR Motivation OR Neophobia OR Body image OR weight concern* OR bias* OR heuristic* OR ((Food OR Product OR Taste) NEAR/10 (Preference* OR Appeal OR Access* OR Concern*)) OR ((Environment* OR Sustainability OR Health OR Diet* OR Food OR Nutritional) NEAR/10 (Awareness OR Value* OR Concern* OR Belief* OR Knowledge OR Literacy OR Attitude* OR Perception* OR Habit*))

OR Residence OR Ethnicity OR Cultur* OR Socio-demographic* OR Gender OR Age OR Education* level OR Income OR ((Socio-economic OR Financial) AND (Status)) OR ((Social OR Peer) NEAR/10 (Norm OR Pressure OR Influence OR Identity OR Support)) OR community influence OR work environment OR public policy

OR Store OR Retail OR Supermarket OR market OR Restaurant OR Take away OR Food Environment OR Neighbourhood OR Proximity OR Availability OR ((Environmental OR Outdoor OR Billboard OR In-store OR POP OR “Point of Purchase”) NEAR/10 (Marketing OR Advert* OR Ads OR Nudge*)) OR ((Food OR Product) AND (Appearance OR Taste OR Convenience)) OR ((On-pack OR Front-of-Pack* OR Packag*) NEAR/10 (Sustainab* OR Health OR Eco* OR Environment* OR Carbon OR “Carbon footprint” OR Organic OR free-range OR “UTZ certified” OR “B Corp” OR non-GMO OR “animal welfare” OR recycling OR Nutrition*) NEAR/10 (Claim* OR Cue* OR Label* OR Certificat* OR Indicat* OR Symbol* OR Table*)) OR Packaging OR Packag* material OR ((Brand OR product) AND (Logo OR Baseline OR Name)) OR Colo$r OR Image OR Photo OR Visual OR Cartoon OR Language OR Price OR Pricing OR Promotion OR Endorser OR Role Model OR Celebrit* OR Influencer

OR Television OR Movie* OR Series OR Internet OR Website* OR Site* OR Social media OR ((Online OR Digital OR Mobile OR Web-based) NEAR/10 (“Food delivery platforms” OR “Grocery shopping” OR Retail OR “Food vendors” OR “Food marketing” OR Application* OR Apps OR Platform OR Communit* OR Marketing OR Advert* OR Ads OR Advergame*)) OR ((Food) AND (Media OR Content OR Post OR Blog)) OR podcast OR user-generated content OR online interaction* OR digital engagement OR e-commerce ))

AND TS=(Zero-waste OR low environmental impact OR Eco-friendly OR Environment-friendly OR Ecologic* OR Green OR Plant-based OR Sustainab* OR Organic OR Seasonal OR Local sourcing OR fair compensation OR fair labo$r OR food equity OR food justice OR food sovereignty OR Fair-trade))

AND TI=(((Food OR Diet* OR Snack OR Drink OR Beverage OR Meal OR Grocer* OR Meat OR Plant-based OR Fruit* OR Vegetable* OR Sustainab* OR Health* OR Nutritious OR Nutrient-dense) AND ( Behavio$r OR Consum* OR Select* OR Eat* OR Intake* OR Choice* OR Chose* OR Perception* OR Attitude* OR Purchas* OR Waste OR food recovery))))

NOT TI=(Bacteri* OR hiv OR Diabet* OR Cancer OR Insulin OR Pharmaceutical OR antimicrob* OR Pregnant OR in vitro OR in vivo OR cardiovascular OR neurological OR autoimmune OR antiviral)) AND LA=(English)

https://www.webofscience.com/wos/woscc/summary/3b644d33-2d0e-4279-8bd3-714a98c37d91-d9979180/relevance/1

PubMed:

(((((Human[Title/Abstract] OR Consumer*[Title/Abstract] OR Adult*[Title/Abstract] OR People[Title/Abstract] OR Senior*[Title/Abstract] OR Man[Title/Abstract] OR Men[Title/Abstract] OR Woman[Title/Abstract] OR Women[Title/Abstract] OR Child*[Title/Abstract] OR Teen*[Title/Abstract] OR Student*[Title/Abstract] OR Adolescent*[Title/Abstract] OR Young[Title/Abstract] OR Boy*[Title/Abstract] OR Girl*[Title/Abstract] OR "Gen X"[Title/Abstract] OR "Gen Y"[Title/Abstract] OR "Gen Z"[Title/Abstract] OR Vulnerable[Title/Abstract] OR low-income[Title/Abstract] OR Marginalized[Title/Abstract] OR Disadvantaged[Title/Abstract] OR Elderly[Title/Abstract] OR immigrant*[Title/Abstract] OR refugee*[Title/Abstract] OR ethnic[Title/Abstract] OR minority[Title/Abstract] OR Indigenous[Title/Abstract] OR unemployed[Title/Abstract] OR customer[Title/Abstract] OR individual[Title/Abstract] OR family[Title/Abstract] OR household[Title/Abstract] OR father*[Title/Abstract] OR mother*[Title/Abstract] OR parent*[Title/Abstract]) AND (Character*[Title/Abstract] OR "Emotional state"[Title/Abstract] OR "Personal conviction*"OR "Political preference"[Title/Abstract] OR Motivation[Title/Abstract] OR Neophobia[Title/Abstract] OR Body image[Title/Abstract] OR weight concern*[Title/Abstract] OR bias*[Title/Abstract] OR heuristic*[Title/Abstract] OR ((Food[Title/Abstract] OR Product[Title/Abstract] OR Taste) AND (Preference[Title/Abstract] OR Appeal[Title/Abstract] OR Access*[Title/Abstract] OR Concern[Title/Abstract])) OR ((Environment*[Title/Abstract] OR Sustainability[Title/Abstract] OR Health[Title/Abstract] OR Diet*[Title/Abstract] OR Food[Title/Abstract] OR Nutritional) AND (Awareness[Title/Abstract] OR Value*[Title/Abstract] OR Concern[Title/Abstract] OR Belief*[Title/Abstract] OR Knowledge[Title/Abstract] OR Literacy[Title/Abstract] OR Attitude*[Title/Abstract] OR Perception*[Title/Abstract] OR Habit*[Title/Abstract])) OR Residence[Title/Abstract] OR Ethnicity[Title/Abstract] OR Cultur*[Title/Abstract] OR Socio-demographic*[Title/Abstract] OR Gender[Title/Abstract] OR Age[Title/Abstract] OR Education* level[Title/Abstract] OR Income[Title/Abstract] OR ((Socio-economic[Title/Abstract] OR Financial[Title/Abstract]) AND (Status[Title/Abstract])) OR ((Social[Title/Abstract] OR Peer) AND (Norm[Title/Abstract] OR Pressure[Title/Abstract] OR Influence[Title/Abstract] OR Identity[Title/Abstract] OR Support[Title/Abstract])) OR "community influence"[Title/Abstract] OR "work environment"[Title/Abstract] OR public policy[Title/Abstract] OR Store[Title/Abstract] OR Retail[Title/Abstract] OR Supermarket[Title/Abstract] OR market[Title/Abstract] OR Restaurant[Title/Abstract] OR Take away[Title/Abstract] OR Food Environment[Title/Abstract] OR Neighbourhood[Title/Abstract] OR Proximity[Title/Abstract] OR Availability[Title/Abstract] OR ((Environmental[Title/Abstract] OR Outdoor[Title/Abstract] OR Billboard[Title/Abstract] OR In-store[Title/Abstract] OR POP[Title/Abstract] OR "Point of Purchase") AND (Marketing[Title/Abstract] OR Advert*[Title/Abstract] OR Ads[Title/Abstract] OR Nudge*[Title/Abstract])) OR ((Food[Title/Abstract] OR Product[Title/Abstract]) AND (Appearance[Title/Abstract] OR Taste[Title/Abstract] OR Convenience[Title/Abstract])) OR ((On-pack[Title/Abstract] OR Front-of-Pack*[Title/Abstract] OR Packag*) AND (Sustainab*[Title/Abstract] OR Health[Title/Abstract] OR Eco*[Title/Abstract] OR Environment*[Title/Abstract] OR Carbon[Title/Abstract] OR "Carbon footprint"[Title/Abstract] OR Organic[Title/Abstract] OR free-range[Title/Abstract] OR "UTZ certified"[Tiab:~0] OR "B Corp"[Title/Abstract] OR non-GMO[Title/Abstract] OR "animal welfare"[Title/Abstract] OR recycling[Title/Abstract] OR Nutrition*) AND (Claim*[Title/Abstract] OR Cue[Title/Abstract] OR Label*[Title/Abstract] OR Certificat*[Title/Abstract] OR Indicat*[Title/Abstract] OR Symbol*[Title/Abstract] OR Table*[Title/Abstract])) OR Packaging[Title/Abstract] OR Packag* material[Title/Abstract] OR ((Brand[Title/Abstract] OR product[Title/Abstract]) AND (Logo[Title/Abstract] OR Baseline[Title/Abstract] OR Name[Title/Abstract])) OR Color[Title/Abstract] OR Colour[Title/Abstract] OR Image[Title/Abstract] OR Photo[Title/Abstract] OR Visual[Title/Abstract] OR Cartoon[Title/Abstract] OR Language[Title/Abstract] OR Price[Title/Abstract] OR Pricing[Title/Abstract] OR Promotion[Title/Abstract] OR Endorser[Title/Abstract] OR Role Model[Title/Abstract] OR Celebrit*[Title/Abstract] OR Influencer[Title/Abstract] OR Television[Title/Abstract] OR Movie*[Title/Abstract] OR Series[Title/Abstract] OR Internet[Title/Abstract] OR Website*[Title/Abstract] OR Site*[Title/Abstract] OR Social media[Title/Abstract] OR ((Online[Title/Abstract] OR Digital[Title/Abstract] OR Mobile[Title/Abstract] OR Web-based) AND ("Food delivery platforms"[Title/Abstract] OR "Grocery shopping"[Title/Abstract] OR Retail[Title/Abstract] OR "Food vendors"[Title/Abstract] OR "Food marketing"[Title/Abstract] OR Application*[Title/Abstract] OR Apps[Title/Abstract] OR Platform[Title/Abstract] OR Communit*[Title/Abstract] OR Marketing[Title/Abstract] OR Advert*[Title/Abstract] OR Ads[Title/Abstract] OR Advergame*[Title/Abstract])) OR ((Food[Title/Abstract]) AND (Media[Title/Abstract] OR Content[Title/Abstract] OR Post[Title/Abstract] OR Blog[Title/Abstract])) OR podcast[Title/Abstract] OR user-generated content[Title/Abstract] OR "online interaction*"[Title/Abstract] OR "digital engagement"[Title/Abstract] OR e-commerce[Title/Abstract])) AND (Zero-waste[Title/Abstract] OR "low environmental impact"[Title/Abstract] OR Eco-friendly[Title/Abstract] OR Environment-friendly[Title/Abstract] OR Ecologic*[Title/Abstract] OR Green[Title/Abstract] OR Plant-based[Title/Abstract] OR Sustainab*[Title/Abstract] OR Organic[Title/Abstract] OR Seasonal[Title/Abstract] OR Local sourcing[Title/Abstract] OR fair compensation[Title/Abstract] OR fair labor[Title/Abstract] OR fair labour[Title/Abstract] OR food equity[Title/Abstract] OR "food justice"[Title/Abstract] OR "food sovereignty"[Title/Abstract] OR Fair-trade[Title/Abstract])) AND (((Food[Title] OR Diet*[Title] OR Snack[Title] OR Drink[Title] OR Beverage[Title] OR Meal[Title] OR Grocer*[Title] OR Meat[Title] OR Plant-based[Title] OR Fruit*[Title] OR Vegetable*[Title] OR Sustainab*[Title] OR Health*[Title] OR Nutritious[Title] OR Nutrient-dense[Title]) AND (Behaviour[Title] OR Behavior[Title] OR Consum*[Title] OR Select*[Title] OR Eating[Title] OR Intake*[Title] OR Choice*[Title] OR Chose*[Title] OR Perception*[Title] OR Attitude*[Title] OR Purchas*[Title] OR Waste[Title] OR food recovery[Title])))) NOT (Bacteri*[Title] OR hiv[Title] OR Diabet*[Title] OR Glucose Metabolism Disorder[MeSH] OR Cancer[Title] OR Neoplasms[MeSH] OR Insulin[Title] OR Pharmaceutical[Title] OR antimicrob*[Title] OR Microbiota[MeSH] OR Pregnant[Title] OR Pregnant Woman[MeSH] OR in vitro[Title] OR in vivo[Title] OR cardiovascular[Title] OR neurological[Title] OR autoimmune[Title] OR antiviral[Title])) NOT (Systematic review[Filter] OR Review [Filter] OR Meta-analysis[Filter]) AND English[Filter])

(((((Human[Title/Abstract] OR Consumer*[Title/Abstract] OR Adult*[Title/Abstract] OR People[Title/Abstract] OR Senior*[Title/Abstract] OR Man[Title/Abstract] OR Men[Title/Abstract] OR Woman[Title/Abstract] OR Women[Title/Abstract] OR Child*[Title/Abs - Search Results - PubMed (nih.gov)

Scopus:

(TITLE-ABS-KEY(Human OR Consumer* OR Adult* OR People OR Senior* OR Man OR Men OR Woman OR Women OR Child* OR Teen* OR Student* OR Adolescen* OR Young OR Boy* OR Girl* OR "Gen X" OR "Gen Y" OR "Gen Z" OR Vulnerable OR low-income OR Marginalized OR Disadvantaged OR Elderly OR immigrant* OR refugee* OR ethnic OR minority OR Indigenous OR unemployed OR customer* OR individual OR family OR household OR father* OR mother* OR parent*))

AND ( TITLE-ABS-KEY(Character* OR “Emotional state” OR "Personal conviction*" OR “Political preference*” OR Motivation OR Neophobia OR “Body image” OR "weight concern*" OR bias* OR heuristic* OR ((Food OR Product OR Taste) W/10 (Preference* OR Appeal OR Access* OR Concern*)) OR ((Environment* OR Sustainability OR Health OR Diet* OR Food OR Nutritional) W/10 (Awareness OR Value* OR Concern* OR Belief* OR Knowledge OR Literacy OR Attitude* OR Perception* OR Habit*))

OR Residence OR Ethnicity OR Cultur* OR Socio-demographic* OR Gender OR Age OR Education* level OR Income OR ((Socio-economic OR Financial) AND (Status)) OR ((Social OR Peer) W/10 (Norm OR Pressure OR Influence OR Identity OR Support)) OR "community influence" OR "work environment" OR "public policy"

OR Store OR Retail OR Supermarket OR market OR Restaurant OR Take away OR "Food Environment" OR Neighbourhood OR Proximity OR Availability OR ((Environmental OR Outdoor OR Billboard OR In-store OR POP OR "Point of Purchase") W/10 (Marketing OR Advert* OR Ads OR Nudge*)) OR ((Food OR Product) W/10 (Appearance OR Taste OR Convenience)) OR ((On-pack OR Front-of-Pack* OR Packag*) W/10 (Sustainab* OR Health OR Eco* OR Environment* OR Carbon OR "Carbon footprint" OR Organic OR free-range OR "UTZ certified" OR "B Corp" OR non-GMO OR "animal welfare" OR recycling OR Nutrition*) W/10 (Claim* OR Cue* OR Label* OR Certificat* OR Indicat* OR Symbol* OR Table*)) OR Packaging OR Packag* material OR ((Brand OR product) W/10 (Logo OR Baseline OR Name)) OR Colo*r OR Image OR Photo OR Visual OR Cartoon OR Language OR Price OR Pricing OR Promotion OR Endorser OR Role Model OR Celebrit* OR Influencer

OR Television OR Movie* OR Series OR Internet OR Website* OR Site* OR Social media OR ((Online OR Digital OR Mobile OR Web-based) W/10 ("Food delivery platforms" OR "Grocery shopping" OR Retail OR "Food vendors" OR "Food marketing" OR Application* OR Apps OR Platform OR Communit* OR Marketing OR Advert* OR Ads OR Advergame*)) OR ((Food) W/10 (Media OR Content OR Post OR Blog)) OR podcast OR "user-generated content" OR online interaction* OR "digital engagement" OR e-commerce) )

AND (TITLE-ABS-KEY(Zero-waste OR "low environmental impact" OR Eco-friendly OR Environment-friendly OR Ecologic* OR Green OR Plant-based OR Sustainab* OR Organic OR Seasonal OR "Local sourcing" OR "fair compensation" OR fair labo*r OR "food equity" OR "food justice" OR "food sovereignty" OR Fair-trade) )

AND ( TITLE( ((Food* OR Diet* OR Snack* OR Drink* OR Beverage* OR Meal* OR Grocer* OR Meat OR Plant-based OR Fruit* OR Vegetable* OR Sustainab* OR Health* OR Nutritious OR Nutrient-dense) AND (Behavio*r OR Consum* OR Select* OR Eat* OR Intake* OR Choice* OR Chose* OR Perception* OR Attitude* OR Purchas* OR Waste OR "food recovery")) ) )

AND NOT ( TITLE( Bacteri* OR hiv OR Diabet* OR Cancer OR Insulin OR Pharmaceutical OR antimicrob* OR Pregnant OR in vitro OR in vivo OR cardiovascular OR neurological OR autoimmune OR antiviral))
