# Supplementary material for: Byte into sustainability: a scoping review of digital food environment attributes that shape consumers’ sustainability perceptions, attitudes, intentions, and behaviours
Source: Int J Behav Nutr Phys Act. 2025 Oct 27;22:132. doi: 10.1186/s12966-025-01832-6 (PMC12560354; doi:10.1186/s12966-025-01832-6)
Supplement: Supplementary file 3 — Supplementary Material 3. [file 12966_2025_1832_MOESM3_ESM.docx]

| **Title** | **Authors** | **Year of Publication** | **Country** | **Study design** | **Medium examined** | **Digital attributes examined** |
| --- | --- | --- | --- | --- | --- | --- |
| Can opinion leaders through Instagram influence organic food purchase behaviour in Saudi Arabia? | Al-Harbi and Badawi | 2021 | Saudi-Arabia | Quantitative | Influencer endorsement on social media | Benevolence of influencer  Integrity of influencer |
| Indulgent or informative logos? Effects on university students’ intention to purchase healthy and sustainable food | Andreani et al. | 2023 | Italy | Quantitative | Food e-commerce platform | informative and indulgent food logo’s |
| The role of social media in consumers’ intentions to buy green food: evidence from Türkiye | Armcutu et al. | 2023 | Turkey | Quantitative | Social media marketing | The presence of social marketing content |
| E-Consumers and Local Food Products: A Perspective for Developing Online Shopping for Local Goods in Poland | Barska and Wojciechowska-Solis | 2020 | Poland | Quantitative | Food e-commerce platform | Convenience of the platform: time saving, home delivery, choosing delivery time  Availability on the platform: wide assortiment, unique products  Managing orders on a daily basis  Being able to correct data  Affiliate program in the app  No returns accepted |
| Social norm-based gamification to promote eco-friendly food choice | Berger | 2019 | Switzerland | Quantitative | Food e-commerce platform | Social norm based feedback (descriptive or injunctive)  Combined social norms (descriptive and injunctive)  Gamified social norm based feedback |
| Effects of a documentary on consumer perception of the environmental impact of meat consumption | Bschaden et al. | 2020 | Germany | Quantitative | Documentary | Documentary on sustainable food related theme |
| Analysing acculturation to sustainable food consumption behaviour in the social media through the lens of information diffusion | Choudhary et al. | 2019 | United Kingdom | Qualitative | Social media influencer endorsement and user-generated content | Influencers sharing content about sustainable food  User-generated content about sustainable food |
| Does online chatter matter for consumer behaviour? A priming experiment on organic food | Danner and Thøgersen | 2022 | Germany | Quantitative | Food e-commerce platform | Priming of high online salient topics |
| A combined Nutri-Score and ‘Eco-Score’ approach for more nutritious and more environmentally friendly food choices? Evidence from a consumer experiment in Belgium | De Bauw et al. | 2021 | Belgium | Quantitative | E-grocery | Combined Nutri-Score and Eco-Score |
| Digital nudges to stimulate healthy and pro-environmental food choices in E-groceries | De Bauw et al. | 2022 | Belgium | Quantitative | E-grocery | Nutri & Eco-scores  Recommendation agents |
| Nudging sustainable consumption: The use of descriptive norms to promote a minority behavior in a realistic online shopping environment | Demarque et al. | 2015 | France | Quantitative | E-grocery | Norms |
| The Sustainability of Fresh Agricultural Produce Live Broadcast Development: Influence on Consumer Purchase Intentions Based on Live Broadcast Characteristics | Guo et al. | 2022 | China | Quantitative | e-commerce live broadcasting | Live broadcast visibility, authenticity, interactivity, entertainment |
| The effectiveness of animal welfare-, environmental-, and health-focused video appeals on implicit and explicit wanting of meat and intentions to reduce meat consumption | Herchenroeder | 2023 | United States | Quantitative | Documentary | Documentary with sustainability theme |
| Social media mediated interaction with peers, experts and anonymous authors: Conversation partner and message framing effects on risk perception and sense-making of organic food | Hilverda et al. | 2017 | The Netherlands | Quantitative | Interactions on social media | Conversation partner in social media interactions  Message frame of messages in social media interactions |
| The Effect of Online Social Proof Regarding Organic Food: Comments and Likes on Facebook | Hilverda et al. | 2018 | The Netherlands | Quantitative | Facebook | Comment valence  Likes of comments |
| How Blockchain-Enabled Drivers Stimulate Consumers’ Organic Food Purchase Intention: An Integrated Framework of Information Systems Success Model Within Stimulus-Organism-Response Theory in the Context of Vietnam | Ho et al. | 2024 | Vietnam | Quantitative | Blockchain technology | Blockchain-enabled traceability  Blockchain-enabled transparency |
| Connecting Small-Scale Producers and Consumers: Exploring the Feasibility of Online Food Hubs in Low-Income Communities | Kaiser et al. | 2020 | United States | Qualitative | Online food hub | Convenience of food hub  Transparency of online vendor  Availability of food on food hub: not being able to touch the food  Product information on food hub |
| Momentum for organic food purchase intention and actual adoption- moderating effects of social media influencer and celebrity endorser | Kalam et al. | 2024 | Bangladesh | Quantitative | Endorsements on social media by influencers and celebrities | Celebrity endorsement  Social media influencer endorsement |
| Making the carbon basket count: Goal setting promotes sustainable consumption in a simulated online supermarket | Kanay et al. | 2021 | France | Quantitative | E-grocery | Goal-setting  Information regarding a product’s sustainability |
| Encouraging Organic Food Consumption through Visualization of Personal Shopping Data | Katzeff et al. | 2020 | Sweden | Qualitative | Shopping dtaa visualisation app | Visualisation of personal shopping data |
| The matching effect of local food and color on ethical dining behaviors: the roles of credibility and green image | Kim et al. | 2023 | Missing | Quantitative | Social media marketing | The presence of social media marketing content  The colours used in the social media marketing content |
| The Influence of Website Quality on Cognitive and Affective Attitudes Towards Organic Food | Kocic et al. | 2022 | Serbia | Quantitative | Food e-commerce platform | Website usability  Website design  Information quality on website |
| Effect of information on public perception of organic foods: a case study | Koswatta et al. | 2022 | United States | Quantitative | User-generated social media content | News on sustainable food delivered via Youtube video |
| Inspiration or risk? How social media marketing of plant-based meat affects young people’s purchase intention | Li et al. | 2022 | China | Quantitative | Social media marketing | Social media marketing  Social media marketing intensity |
| The effect of social media marketing on consumers' purchase intention of organic food: the role of perceived value, trust and social identity | Lian et al. | 2023 | Malaysia | Quantitative | Social media marketing | Informative value of social media marketing content  Interaction with social media marketing content  e-WOM |
| Factors influencing consumers’ continuous purchase intention on fresh food e-commerce platforms: An organic foods-centric empirical investigation | Lin et al. | 2021 | China | Quantitative | Food e-commerce platform | Platform characteristics |
| Purchasing organic food with social commerce: An integrated food-technology consumption values perspective | Lin et al. | 2020 | China | Quantitative | Social commerce | Interactivity of platform  Availability of recommendations and feedback on platform |
| Media influences on consumption trends: Effects of the film Food, Inc. on organic food sales in the U.S | Ma et al. | 2020 | United States | Quantitative | Documentary | Documentary about sustainability theme |
| Determinants of Green Smartphone Application Adoption for Sustainable Food Consumption Among University Students | Matin et al. | 2024 | Georgia | Quantitative | Green smartphone application | Timeliness of app  Relevance of app  Sufficiency of app  Customisation of app |
| Stimulating Sustainable Food Choices Using Virtual Reality: Taking an Environmental vs Health Communication Perspective on Enhancing Response Efficacy Beliefs | Meijers et al. | 2022 | The Netherlands | Quantitative | VR grocery | impact messages |
| Ninety Minutes to Reduce One's Intention to Eat Meat: A Preliminary Experimental Investigation on the Effect of Watching the Cowspiracy Documentary on Intention to Reduce Meat Consumption | Pabian et al. | 2020 | Belgium | Quantitative | Documentary | Documentary with sustainability theme |
| Which Determinants Impact Consumer Purchase Behavior toward Online Purchasing of Organic Food Products? | Pahari et al. | 2023 | India | Quantitative | Food e-commerce platform | Product information on platform  Accessibility of platform |
| Effects of environmental impact labels on the sustainability of food purchases: A randomised controlled trial in an experimental online supermarket | Potter et al. | 2024 | United Kingdom | Quantitative | E-grocery | Ecolabels |
| Swipe to Sustain: Exploring Consumer Behaviors in Organic Food Purchasing via Instagram Social Commerce | Poureisa et al. | 2024 | Iran | Quantitative | Social commerce | Availability of recommendations and referrals on social commerce platform  Availability of reviews and ratings on social commerce platform  Social media influencer endorsement on social commerce platform |
| Consumption Value and Organic Food Purchasing Behavior of Online Consumers | Prince et al. | 2024 | Bangladesh | Quantitative | Food e-commerce wensite | Website quality |
| Factors influencing young adults’ organic food purchase intention on fresh food e-commerce platforms | Qi et al. | 2024 | China | Quantitative | Food e-commerce platform | System quality of e-commerce platform  Service quality of e-commerce platform  Delivery system of e-commerce platform  Evaluation system of e-commerce platform |
| Organic and online attributes for buying and selling agricultural products in the e-marketplace in Spain | Robina-Ramírez et al. | 2020 | Spain | Quantitative | Organic food e-marketplace | Ease of browsing of the platform  Reliable access of the platform |
| Using Virtual Reality to Stimulate Healthy and Environmentally Friendly Food Consumption among Children: An Interview Study | Smit et al., | 2021 | The Netherlands | Qualitative | VR supermarket | Textual and visual environmental or health messages |
| Exploratory modelling and ranking of the trust factors of messages about organic foods in social networks | Sobhanifard and Eshtiaghi | 2020 | Iran | Quantitative | Social media marketing | Social media marketing focused on valid experiences, valid guarantees and certifications, valid information on product benefits, naturalness, communication with previous buyers |
| How Do Fresh Live Broadcast Impact Consumers’ Purchase Intention? Based on the SOR Theory | Song et al. | 2022 | China | Quantitative | Live e-commerce broadcast | Visibility in live broadcast  Interactivity in live broadcast  Authenticity of live broadcast |
| The impact of a blockchain-based food traceability system on the online purchase intention of organic agricultural products | Tao et al. | 2024 | China | Quantitative | Blockchain technology | Blockchain-based food traceability system |
| Organic food consumerism through social commerce in China | Tariq et al. | 2019 | United States | Quantitative | Social commerce | Visual appeal of website  Navigation design of website  Informative value of website |
| A Modeling for Enhancing Consumer Trust in Organic Food through Authentic Content in Social Networks | Tashakkori et al. | 2023 | Iran | Quantitative | Social media marketing | Social media marketing content focused on standards  Social media marketing content focused on product validity  Social media marketing focused on credible recommenders (driver)  Social media marketing content focused on valid supply |
| Nudging plant-based alternatives to meat and dairy in a real-life online supermarket: A randomized controlled trial | van der Vliet et al. | 2024 | The Netherlands | Quantitative | E-grocery | Combination of nudging strategies |
| Can Product Information Steer towards Sustainable and Healthy Food Choices? A Pilot Study in an Online Supermarket | van der Waal et al. | 2022 | The Netherlands | Quantitative | E-grocery | Explanatory sustainability claim |
| Intervention strategies to promote healthy and sustainable food choices among parents with lower and higher socioeconomic status | Vos et al. | 2022 | Belgium | Qualitative | App as sustainable food guide  E-commerce platform  Social media content | Information and inspiration  User friendliness  Time saving features  Online meal schedules  Online eco and nutri labels  Online shopping lists  Automatisation of ingredient adding  Convenience of online application  Free app  Filter function  Easy ordering of online meal boxes  No flexible geographical availability  Social media as inspiration  Lack of flexible pick-up times |
| The antecedents of customer satisfaction in the live-streaming commerce of green agricultural products | Wang et al. | 2024 | China | Quantitative | E-commerce live broadcasting | Commodities in live broadcasting  Livestreaming contents  Supporting services of broadcast |
| Mobile apps as a sustainable shopping guide: The effect of eco-score rankings on sustainable food choice | Weber | 2021 | Germany | Quantitative | Mobile app | Eco-rankings |
| Comparison of Consuming Habits on Organic Food—Is It the Same? Hungary Versus China | Wu and Takács-György | 2022 | Hungary, China | Quantitative | Social media content | Social media use  Social media marketing |
| Do green information transparency and exposure always boost online sales of organic food? An Evidence from China | Wu et al. | 2024 | China | Quantitative | Food e-commerce platform | Green information transparency of e-commerce platform |
| The effect of social media influencer marketing on sustainable food purchase: Perspectives from multi-group SEM and ANN analysis | Wu et al. | 2023 | China | Quantitative | Social media influencer marketing | Intimate self-disclosure of influencer  Environmental concerns of influencer |
| ‘I'm like you, and I like what you like’ sustainable food purchase influenced by vloggers: A moderated serial-mediation model | Xu et al. | 2021 | China | Quantitative | Influencer vlogs | Parasocial interaction between vloggers and audience  Audience participation on influencer vlogs  Product-vlogger congruence |
| Consumers’ Purchase Intention of Organic Food via Social Media: The Perspectives of Task-Technology Fit and Post-acceptance Model | You et al. | 2020 | Missing | Quantitative | Organic food social media forum | Technology characteristics of an organic food social media forum |
| Influence of online product presentation on consumers’ trust in organic food: A mediated moderation model | Yue et al. | 2017 | China | Quantitative | Food e-commerce website | Mediarichness of website  Review length on website |
| Czech Consumers’ Preference for Organic Products in Online Grocery Stores during the COVID-19 Pandemic | Zámková et al. | 2022 | Czech Republic | Quantitative | E-grocery | Convenience of e-grocery: time-saving, ease of searching  Availability of e-grocery: great product choise, fast |
| Examining the Factors That Affect Consumers’ Purchase Intention of Organic Food Products in a Developing Country | Zayed et al. | 2022 | Egypt | Quantitative | Social media | e-Wom |
| Evaluation of the factors affecting consumers’ purchases of fresh food online from China and Hungary | Zhang and Rudnak | 2023 | China, Hungary | Quantitative | Food e-commerce platform | Logistics of the e-commerce platform  Comments on an e-commerce platform |
